# Controlled motivation and physics achievement: a serial mediation analysis through self-efficacy and learning strategies in Chinese high school students

**DOI:** 10.3389/fpsyg.2026.1745451

**Published:** 2026-02-04

**Authors:** Xiao Li, Xue Lin, Jingxian Lin, Mengting Yan

**Affiliations:** Wenling High School, Wenling, Zhejiang, China

**Keywords:** controlled motivation, Gaokao, high-stakes testing, learning strategies, physics achievement, self-efficacy, serial mediation

## Abstract

**Background:**

In high-stakes examination contexts such as China’s Gaokao system, students frequently experience controlled motivation characterized by external pressure and learning anxiety. While research consistently demonstrates that controlled motivation undermines academic achievement, the psychological mechanisms through which this occurs remain underspecified. This study examined whether and how controlled motivation influences physics achievement through sequential effects on self-efficacy beliefs and learning strategy use among Chinese high school students.

**Methods:**

A total of 496 s-year high school students (65.9% male; *M* age = 16.98 years) from three schools in Zhejiang Province completed validated measures of controlled motivation, physics self-efficacy, learning strategies, and physics achievement. Serial mediation analysis using Hayes’ PROCESS Model 6 tested the hypothesized pathways through which controlled motivation influences achievement.

**Results:**

The negative association between controlled motivation and physics achievement operates primarily through psychological and behavioral mechanisms rather than through direct pathways. These findings suggest that interventions targeting self-efficacy enhancement and learning strategy support may effectively buffer against the detrimental effects of external pressure in high-stakes educational contexts. This research integrates self-determination theory, social cognitive theory, and self-regulated learning theory into a unified framework for understanding motivation-achievement relationships.

**Conclusion:**

These findings suggest that interventions targeting self-efficacy enhancement and learning strategy instruction could potentially buffer against the detrimental correlates of external pressure in high-stakes educational contexts. The research contributes to theoretical understanding by demonstrating empirical support for an integrated framework combining self-determination theory, social cognitive theory, and self-regulated learning theory in explaining motivation-achievement relationships.

## 1 Introduction

Physics education serves as a critical gateway to STEM careers, yet high-stakes testing environments often transform learning into a pressure-driven exercise. In China, where physics constitutes a core component of the National College Entrance Examination (Gaokao), students face intense pressure to achieve high scores that directly determine university admission and career prospects (Wang, 1997). This creates a fundamental paradox. While policy initiatives such as the 2024 “STEM Education 2035 Action Plan” emphasize cultivating scientific inquiry and innovation, the examination system incentivizes strategic performance over genuine understanding. Students may master problem-solving techniques sufficient for examination success while lacking conceptual comprehension essential for advanced study. Understanding the psychological mechanisms through which students navigate this high-pressure environment has become urgent for both theoretical advancement and educational reform.

### 1.1 The problem of controlled motivation in high-stakes learning

Self-determination theory distinguishes between autonomous motivation, driven by intrinsic interest and personal values, and controlled motivation, driven by external pressure and anxiety (Klein, 2019). While autonomous motivation consistently predicts positive learning outcomes (Reeve, 2012), controlled motivation presents a troubling picture in high-stakes environments. When students engage with physics primarily to satisfy external demands such as parental expectations or fear of failure rather than intellectual curiosity, several detrimental processes emerge. High-stakes testing environments activate extrinsic motivation while suppressing intrinsic interest, leading students to adopt learning strategies focused on memorization rather than conceptual understanding (Deci and Ryan, 2016). When external rewards and punishments dominate, learning becomes fragmented and instrumental, with students disengaging once immediate incentives are removed (Jeno et al., 2017). In China’s Gaokao system, where most students pursue physics due to examination requirements rather than genuine interest, this predominance of controlled motivation may paradoxically undermine the achievement outcomes it ostensibly serves.

Previous research has predominantly examined how autonomous motivation enhances achievement, leaving the mechanisms through which controlled motivation impairs performance inadequately understood. Jiaxia et al. (2000) provided early evidence that controlled motivation correlates negatively with academic performance in Chinese students, yet the psychological pathways mediating this relationship remain unexplored. The current study addresses this gap by examining controlled motivation operationalized through learning anxiety and external pressure, where higher scores indicate greater motivational problems and psychological distress.

### 1.2 Self-efficacy: from beliefs to performance

Self-efficacy, defined as domain-specific beliefs in one’s capability to execute actions required for specific performance attainments (Bandura et al., 1999), represents a distinct psychological mechanism linking motivation to achievement. Unlike global confidence, self-efficacy is task-oriented and context-dependent (Yokoyama, 2019), making it particularly relevant for understanding physics learning. Students with strong physics self-efficacy believe they can master challenging concepts and succeed on demanding assessments, whereas those with weak self-efficacy doubt their capabilities even when competent.

Empirical evidence consistently demonstrates that physics self-efficacy predicts academic performance, with meta-analytic effect sizes ranging from medium to large (Honicke and Broadbent, 2016). However, the relationship between controlled motivation and self-efficacy likely operates through distinct processes. When learning stems from external pressure rather than interest, heightened anxiety interferes with performance and erodes confidence, success becomes attributed to external factors rather than personal capability, and the absence of intrinsic satisfaction reduces the reinforcing effect of achievement on efficacy beliefs. Bandura et al. (1999) proposed that motivational quality shapes whether individuals develop robust or fragile confidence, yet this proposition has received limited empirical testing in high-stakes educational contexts dominated by controlled motivation.

### 1.3 Learning strategies: the behavioral translation of beliefs

A crucial question concerns how self-efficacy beliefs translate into actual academic performance. Self-regulated learning theory suggests that self-efficacy influences achievement substantially through its effects on learning strategy selection and use (Torrano Montalvo and González Torres, 2004). Students with strong efficacy beliefs employ sophisticated cognitive and metacognitive strategies including elaboration, organization, and self-monitoring. They persist through challenging tasks, viewing difficulties as surmountable. Conversely, students with weak efficacy beliefs adopt surface learning strategies characterized by rote memorization and procedural reproduction, avoiding difficult problems and disengaging when obstacles arise.

In physics education specifically, this distinction proves particularly consequential. Mastering physics requires understanding abstract concepts, establishing connections between mathematical formalism and physical intuition, and applying principles flexibly to novel situations. These demands necessitate deep learning strategies. Surface approaches focused on formula memorization prove inadequate for genuine comprehension and flexible application. The Learning and Study Strategies Inventory for High School students (LASSI-HS) captures this distinction through dimensions including information processing, concentration, and self-testing. The Chinese version (LASSI-HS-CV) provides a validated measure of learning strategy quality specifically adapted for Chinese contexts (Taheri et al., 2017).

### 1.4 The chain from pressure to performance: a serial mediation framework

Integrating self-determination theory, social cognitive theory, and self-regulated learning theory suggests a serial mediation model wherein controlled motivation influences achievement through sequential effects on self-efficacy and learning strategies. This theoretical integration proposes a specific causal sequence: external pressure and anxiety (controlled motivation) undermine students’ confidence in their capabilities (self-efficacy), weakened confidence leads to adoption of ineffective learning approaches (surface strategies), and surface strategy use ultimately results in poor performance. Each link represents a distinct mechanism operating sequentially to produce cumulative effects.

The theoretical logic underlying each pathway merits specification. First, controlled motivation negatively influences self-efficacy through multiple processes. External pressure triggers performance anxiety that interferes with task execution and creates negative mastery experiences. Controlled motivational orientations promote external attributions for success and internal attributions for failure, patterns that systematically undermine efficacy development. Second, self-efficacy influences learning strategy use because students with strong efficacy beliefs invest greater cognitive effort, actively engage with challenging material, and employ sophisticated strategies. Students with weak beliefs minimize cognitive investment to protect fragile self-concepts. Third, learning strategies directly influence achievement through their effects on cognitive processing depth.

Prior research has examined these relationships primarily in isolation, documenting bivariate correlations while leaving the complete pathway untested. Testing this serial mediation model addresses three critical gaps: moving beyond correlation to examine causal sequences, examining controlled motivation’s detrimental pathways where such motivation predominates, and focusing on high school physics learning in high-stakes contexts, an understudied yet educationally consequential domain.

### 1.5 Present study

We investigated the serial mediation pathway from controlled motivation through self-efficacy and learning strategies to physics achievement among Chinese high school students preparing for the Gaokao examination. This high-stakes context provides an ideal setting for studying controlled motivation’s effects, as students experience intense external pressure from multiple sources.

We hypothesized that controlled motivation influences physics achievement both directly and indirectly through sequential effects on self-efficacy and learning strategies. Specifically, we predicted that controlled motivation would negatively correlate with self-efficacy, learning strategies, and achievement; self-efficacy would positively correlate with learning strategies and achievement; and the indirect effect through the serial pathway of self-efficacy and learning strategies would be statistically significant. Testing this complete model allows identification of specific mechanisms linking motivation to achievement, providing both theoretical insight into how motivational processes operate in high-pressure contexts and practical guidance for educational interventions targeting the most effective leverage points for supporting student success.

## 2 Methods

### 2.1 Participants and sampling procedure

#### 2.1.1 Educational context

This study was conducted in Wenling City, Zhejiang Province, China, during the 2024 fall semester. Wenling’s high schools implement a three-tier academic tracking system typical of Chinese secondary education: advanced classes (top 15% on junior high entrance examination), key classes (15th-50th percentile), and regular classes (remaining students). Students are assigned to tracks based solely on prior academic performance, with limited inter-track mobility. Physics is mandatory for students pursuing science tracks and represents a core Gaokao examination component for STEM university admission.

#### 2.1.2 Sample characteristics

A stratified convenience sampling approach ensured representation across academic levels. Three high schools were purposively selected representing different institutional tiers (one top-tier municipal school, one mid-tier district school, one regular township school). All second-year students (Grade 11) who elected physics as a Gaokao subject were invited to participate. Second-year students were targeted because they have completed 1 year of high school physics, allowing motivational patterns to stabilize, while avoiding extreme examination pressure faced by third-year students.

Of 557 eligible students approached, 496 provided complete data (response rate = 89.1%). Excluded cases (*n* = 61) resulted from incomplete responses (*n* = 43), absence during data collection (*n* = 14), or consent withdrawal (*n* = 4). Chi-square analyses revealed no significant differences between included and excluded participants in gender, *χ^2^* (1) = 0.87, *p* = 0.351, or class type distribution, *χ^2^* (2) = 2.34, *p* = 0.310, suggesting negligible bias from missing data. The final sample comprised 327 male students (65.9%) and 169 female students (34.1%), ages 15–19 years (*M* = 16.98, *SD* = 0.52). The gender distribution reflects documented gender disparities in physics selection in Chinese high schools (He et al., 2020). Regarding academic tracks, 155 students (31.3%) were enrolled in advanced classes, 133 (26.8%) in key classes, and 208 (41.9%) in regular classes. Additionally, 71 students (14.3%) reported attending supplementary physics tutorials.

### 2.2 Measures

#### 2.2.1 Controlled motivation

Physics learning motivation was assessed using an adapted version of the Academic Motivation Scale-Learning in Time (AMSLIT; Schreglmann, 2018). This 21-item scale captures externally-driven academic motivation and learning anxiety in examination-oriented contexts. Unlike traditional motivation measures assessing intrinsic interest, this instrument specifically operationalizes controlled motivation through indicators of external pressure, anxiety, and compulsive study behaviors. Sample items include: “In order to complete physics homework on time, you would rather stay up all night and forget to eat and sleep,” “You feel extremely anxious when physics exam approaches,” and “You study physics primarily because your parents and teachers expect you to do well.”

Responses were recorded using a binary format (1 = does not fit me, 2 = fits me), with total scores ranging from 21 to 42. This binary format was selected to reduce response burden and minimize social desirability bias, as pilot testing indicated that Chinese adolescents responded more candidly to yes/no questions about anxiety and pressure than to graded scales. Critically, higher scores indicate greater controlled motivation, characterized by increased external pressure, anxiety, and problematic motivational patterns. Thus, the scale measures motivation quality rather than intensity, with higher scores reflecting more maladaptive profiles. In the current sample, internal consistency was acceptable (Cronbach’s *α* = 0.758).

#### 2.2.2 Self-efficacy

Physics-specific academic self-efficacy was measured using an adapted Academic Self-Efficacy Scale (ASES; Elias and Loomis, 2000). The original scale was modified to focus specifically on physics learning contexts, ensuring domain-specificity consistent with Bandura’s conceptualization of self-efficacy as task- and context-dependent (Akhmedjanova, 2024). The 23-item scale assesses students’ confidence in performing various physics-related academic tasks. Sample items include: “I think I can grasp the content taught by the teacher in physics class in a timely manner,” “I am confident I can understand the most difficult material presented in physics readings and lectures,” and “I am certain I can figure out how to solve even the most challenging physics problems.” Responses were recorded on a 5-point Likert scale (1 = completely inconsistent with me, 5 = completely consistent with me). Total scores (range: 23–115) were calculated by summing all items, with higher scores indicating stronger physics self-efficacy beliefs. In the current sample, the Chinese physics-specific version demonstrated strong internal consistency (Cronbach’s *α* = 0.867), exceeding conventional thresholds for research applications.

#### 2.2.3 Learning strategies

Learning strategies were assessed using the Chinese version of the Learning and Study Strategies Inventory-High School (LASSI-HS-CV; An et al., 2019). The original LASSI-HS (Eldredge, 1990) is a comprehensive measure of learning and study strategies widely used in educational research. The Chinese version, validated by Teng and Zhang (2016), consists of 28 items assessing multiple dimensions of learning strategy use including information processing, concentration, selecting main ideas, and self-testing strategies. Sample items include: “I use study aids like highlighting, making notes, or creating concept maps to help me learn,” “When the teacher presents new material, I try to identify the main concepts,” and “I try to connect what I am learning to my own experiences.” Responses were recorded on a Likert scale, with total scores computed by summing all items. Higher scores indicate greater use of effective learning strategies. The Chinese version has demonstrated strong psychometric properties in prior research, with total scale reliability of *α* = 0.905 (Wang et al., 2021). In the current sample, internal consistency for the total scale was *α* = 0.889.

#### 2.2.4 Physics academic performance

Physics performance was operationalized as students’ standardized mid-term examination scores administered in fall 2024. These examinations are mandated by Zhejiang Provincial Education Department and administered across all provincial high schools using identical schedules, content coverage, and grading criteria. The examination covers mechanics and thermodynamics content from Grade 11 curriculum, with items including multiple-choice questions (40%), short-answer calculations (40%), and comprehensive application problems (20%) designed to assess conceptual understanding, procedural skill, and problem-solving ability. Scores were obtained through official school administrative records with appropriate institutional permissions. To protect privacy, identifying information was removed before data transfer, with students linked to questionnaire responses through anonymous study ID codes. Scores are reported on a 100-point scale. In the current sample, physics scores ranged from 40 to 100 points (*M* = 85.47, *SD* = 9.87). The distribution showed slight negative skewness (skewness = −0.58, *SE* = 0.11) but did not significantly deviate from normality, D (496) = 0.047, *p* = 0.082, supporting parametric analyses.

### 2.3 Procedure

Data collection occurred in October 2024, 6 weeks into the fall semester and 3 weeks prior to mid-term examinations. This timing allowed sufficient instruction time for motivational patterns to stabilize while minimizing immediate pre-examination anxiety that might artificially inflate controlled motivation scores. All procedures were reviewed and approved by the Ethics Committee of Wenling High School and complied with ethical principles outlined in the Declaration of Helsinki (World Medical Association, 2013). Information letters and consent forms were distributed to all eligible students and parents/guardians 1 week before data collection. Materials explained the voluntary nature of participation, anonymity of responses, absence of consequences for declining, and right to withdraw without penalty. Students were explicitly informed that responses would not be shared with teachers or parents and would not affect academic standing. Written informed consent was obtained from all participants, with parental consent obtained for students under age 18. Questionnaires were administered during regular class periods through the Xuehai System, a digital learning management platform widely used in Zhejiang schools. Data collection occurred simultaneously across all participating classes within each school to minimize cross-contamination. Trained research assistants (advanced graduate students in educational psychology) read standardized instructions emphasizing voluntary participation, confidentiality, and honest responding. Students were informed that there were no correct or incorrect answers.

The digital survey presented items in fixed order: demographic information, controlled motivation scale, self-efficacy scale, and learning strategies scale. Students completed the survey at their own pace, typically within 25–35 min. Research assistants remained present to answer procedural questions but did not provide content guidance. Upon completion, students received a brief debriefing message with research team contact information. Physics examination scores were obtained approximately 4 weeks after questionnaire administration. Scores were extracted from school databases by school personnel and transferred to the research team with all identifying information removed. Data were stored on password-protected servers accessible only to the research team, complying with Chinese data protection regulations and institutional ethics requirements.

### 2.4 Data analysis strategy

Data analysis proceeded through multiple stages using SPSS version 24.0 (IBM Corp., 2016) and the PROCESS macro version 4.0 for SPSS (Hayes, 2022). Prior to primary analyses, data were screened for accuracy, missing values, outliers, and statistical assumption adherence. Missing data were minimal (<2%) and missing completely at random (Little’s MCAR test: *χ^2^* = 47.23, *df* = 52, *p* = 0.654), allowing listwise deletion without bias. Univariate outliers were identified using standardized scores (|*z|* > 3.29), and multivariate outliers using Mahalanobis distance with *χ^2^* critical value at *p* < 0.001. Five cases were identified as multivariate outliers and removed, resulting in the final sample of 496 participants.

Stage 1: Descriptive and Bivariate Analyses. Descriptive statistics were computed for all study variables, including means, standard deviations, ranges, skewness, and kurtosis. Independent samples t-tests examined gender differences. One-way ANOVA tested differences across class types. Pearson correlation coefficients examined bivariate relationships among controlled motivation, self-efficacy, learning strategies, and physics performance. Effect sizes were interpreted using Cohen’s guidelines (small: *r* = 0.10, medium: *r* = 0.30, large: *r* = 0.50).

Stage 2: Serial Mediation Analysis. The hypothesized serial mediation model wherein controlled motivation influences physics performance through sequential effects on self-efficacy and learning strategies was tested using Hayes’ (2022) PROCESS macro-Model 6 for SPSS. This analytical approach allows simultaneous estimation of multiple pathways through which an independent variable influences a dependent variable via two mediators arranged in serial order. The model estimates the total effect of controlled motivation on physics performance (path c), the direct effect controlling for both mediators (path c’), and several specific indirect pathways. The first pathway examines whether controlled motivation influences self-efficacy (path a₁), which in turn affects physics performance (path b₁). The second pathway tests whether controlled motivation directly influences learning strategies (path a₂), which then affect performance (path b₂). Most critically for the current theoretical framework, the serial mediation pathway examines whether controlled motivation influences self-efficacy (path a₁), which subsequently affects learning strategies (path d₂₁), which finally influence physics performance (path b₂). This serial pathway captures the theoretically proposed sequence wherein motivational quality shapes efficacy beliefs, which then influence behavioral strategy selection, culminating in performance outcomes.

Statistical significance of all indirect effects was evaluated using bias-corrected bootstrap confidence intervals based on 10,000 resampled datasets, following current best practices in mediation analysis (Hayes, 2022). Bootstrap procedures are superior to traditional approaches such as Sobel tests because they make no distributional assumptions about indirect effects, which are typically non-normal due to being products of multiple parameters estimates. An indirect effect is considered statistically significant at *α* = 0.05 when its 95% bias-corrected bootstrap confidence interval excludes zero. Serial mediation is supported when the sequential indirect effect (controlled motivation → self-efficacy → learning strategies → physics performance) is statistically significant, indicating that controlled motivation influences performance through its cascading effects on beliefs and behaviors. Partial mediation is indicated when both indirect effects and the direct effect (c’) are significant, whereas complete mediation occurs when indirect effects are significant but the direct effect is not. Effect sizes for mediation pathways were quantified using the proportion of total effect mediated (PM = indirect effect/total effect), indicating the percentage of controlled motivation’s total influence on performance operating through each specific mediational pathway. All significance tests were two-tailed with alpha set at 0.05.

## 3 Results

### 3.1 Preliminary analyses

#### 3.1.1 Sample characteristics

The sociodemographic characteristics of the sample are presented in Table 1. The final sample comprised 496 students, with males representing approximately two-thirds of participants (65.9%, *n* = 327) and females one-third (34.1%, *n* = 169). This gender distribution reflects the well-documented phenomenon that male students disproportionately select physics in Chinese high schools. The majority of students were 17 years old (82.9%, *n* = 411), consistent with typical second-year high school enrollment. The age distribution showed minimal variability (*M* = 16.98, *SD* = 0.52), with ages ranging from 15 to 19 years.

**Table 1:** Sociodemographic characteristics of the sample.

Variable	Category	*N*	%
Gender	Male	327	65.9
	Female	169	34.1
Age	15	5	1.0
	16	43	8.7
	17	411	82.9
	18	31	6.3
	19	6	1.2
Class type	Advanced (Classes 1–4)	155	31.3
	Key (Classes 5–8)	133	26.8
	Regular (Classes 14–20)	208	41.9
After-school tutorials	Yes	71	14.3
	No	425	85.7

N = 496. Advanced classes enroll students scoring in the top 15% on entrance examinations; key classes enroll students in the 15th-50th percentile; regular classes enroll remaining students.

Regarding academic track distribution, 155 students (31.3%) were enrolled in advanced classes (Classes 1–4), representing students who scored in the top 15% on their junior high entrance examinations. An additional 133 students (26.8%) were enrolled in key classes (Classes 5–8), representing the 15th-50th percentile, while 208 students (41.9%) were enrolled in regular classes (Classes 14–20), representing the remaining student population. This distribution approximates the population distribution across track levels in participating schools. A minority of students (14.3%, *n* = 71) reported attending supplementary after-school physics tutorial classes, while the vast majority (85.7%, *n* = 425) did not participate in additional instruction outside regular school hours (see Table 1).

#### 3.1.2 Descriptive statistics

Descriptive statistics for all study variables are presented in Table 2. Controlled motivation scores ranged from 21 to 42 (*M* = 29.34, *SD* = 5.12), with the distribution showing slight positive skewness (skewness = 0.43, *SE* = 0.11), indicating that most students reported moderate levels of external pressure and learning anxiety, though a substantial minority experienced high controlled motivation. Self-efficacy scores ranged from 38 to 113 (*M* = 76.28, *SD* = 14.63), approximating a normal distribution (skewness = −0.21, *SE* = 0.11). Learning strategies scores ranged from 42 to 136 (*M* = 89.45, *SD* = 18.72), also showing approximately normal distribution (skewness = 0.15, *SE* = 0.11). Physics achievement scores, as previously noted, ranged from 40 to 100 (*M* = 85.47, *SD* = 9.87), with slight negative skewness (skewness = −0.58, *SE* = 0.11). All variables fell within acceptable ranges for skewness and kurtosis (|skewness| < 1.0, |kurtosis| < 3.0), supporting the appropriateness of parametric statistical procedures (see Table 2).

**Table 2:** Descriptive statistics for study variables.

Variable	*M*	*SD*	Range	Skewness	Kurtosis	*α*
Controlled motivation	29.34	5.12	21–42	0.43	−0.15	0.758
Self-efficacy	76.28	14.63	38–113	−0.21	0.08	0.867
Learning strategies	89.45	18.72	42–136	0.15	−0.32	0.889
Physics achievement	85.47	9.87	40–100	−0.58	0.43	—

*N* = 496. α = Cronbach’s alpha coefficient.

#### 3.1.3 Gender and class type differences

Independent samples t-tests revealed significant gender differences in self-efficacy, *t*(494) = 3.42, *p* < 0.001, *d* = 0.32, with male students (*M* = 78.15, *SD* = 14.28) reporting higher physics self-efficacy than female students (*M* = 72.84, *SD* = 14.72). No significant gender differences emerged for controlled motivation, *t* (494) = 1.23, *p* = 0.219, *d* = 0.12, learning strategies, *t*(494) = 0.87, *p* = 0.385, *d* = 0.08, or physics achievement, *t*(494) = 1.65, *p* = 0.099, *d* = 0.15.

One-way ANOVA examining differences across class types (advanced, key, regular) revealed significant effects for all variables. Advanced class students reported lower controlled motivation (*M* = 27.42, *SD* = 4.88) compared to key class (*M* = 29.18, *SD* = 5.02) and regular class students (*M* = 30.75, *SD* = 5.15), *F* (2, 493) = 15.34, *p* < 0.001, *η^2^* = 0.059. Advanced class students also demonstrated higher self-efficacy (*M* = 82.56, *SD* = 13.42), *F*(2, 493) = 24.68, *p* < 0.001, *η^2^* = 0.091, more effective learning strategies (*M* = 98.34, *SD* = 17.25), *F*(2, 493) = 18.92, *p* < 0.001, *η^2^* = 0.071, and superior physics achievement (*M* = 91.23, *SD* = 7.45), *F*(2, 493) = 32.47, *p* < 0.001, *η^2^* = 0.116, compared to key and regular class students. *Post-hoc* Tukey HSD tests confirmed significant differences between all three groups for physics achievement (all *p*s < 0.01).

### 3.2 Bivariate correlations

Pearson correlation coefficients among study variables are presented in Table 3. Consistent with theoretical predictions, controlled motivation demonstrated significant negative correlations with self-efficacy (*r* = −0.411, *p* < 0.001), learning strategies (*r* = −0.325, *p* < 0.001), and physics achievement (*r* = −0.279, *p* < 0.001). These negative correlations indicate that students experiencing greater external pressure and learning anxiety reported lower confidence in their physics capabilities, employed less effective learning strategies, and achieved lower examination scores. Self-efficacy showed significant positive correlations with learning strategies (*r* = 0.523, *p* < 0.001) and physics achievement (*r* = 0.367, *p* < 0.001), indicating that students with stronger efficacy beliefs engaged in more sophisticated learning behaviors and performed better academically. Learning strategies also demonstrated a significant positive correlation with physics achievement (*r* = 0.425, *p* < 0.001), confirming that strategy use relates meaningfully to performance outcomes. All correlations were in the hypothesized directions and of moderate to large magnitude according to Cohen’s guidelines, providing initial support for the proposed theoretical relationships (See Table 3).

**Table 3:** Pearson correlations among study variables.

Variable	1	2	3	4
1. Controlled motivation	—			
2. Self-efficacy	−0.411***	—		
3. Learning strategies	−0.325***	0.523***	—	
4. Physics achievement	−0.279***	0.367***	0.425***	—

*N* = 496. ***p < 0.001.

### 3.3 Serial mediation analysis

**Figure 1:**
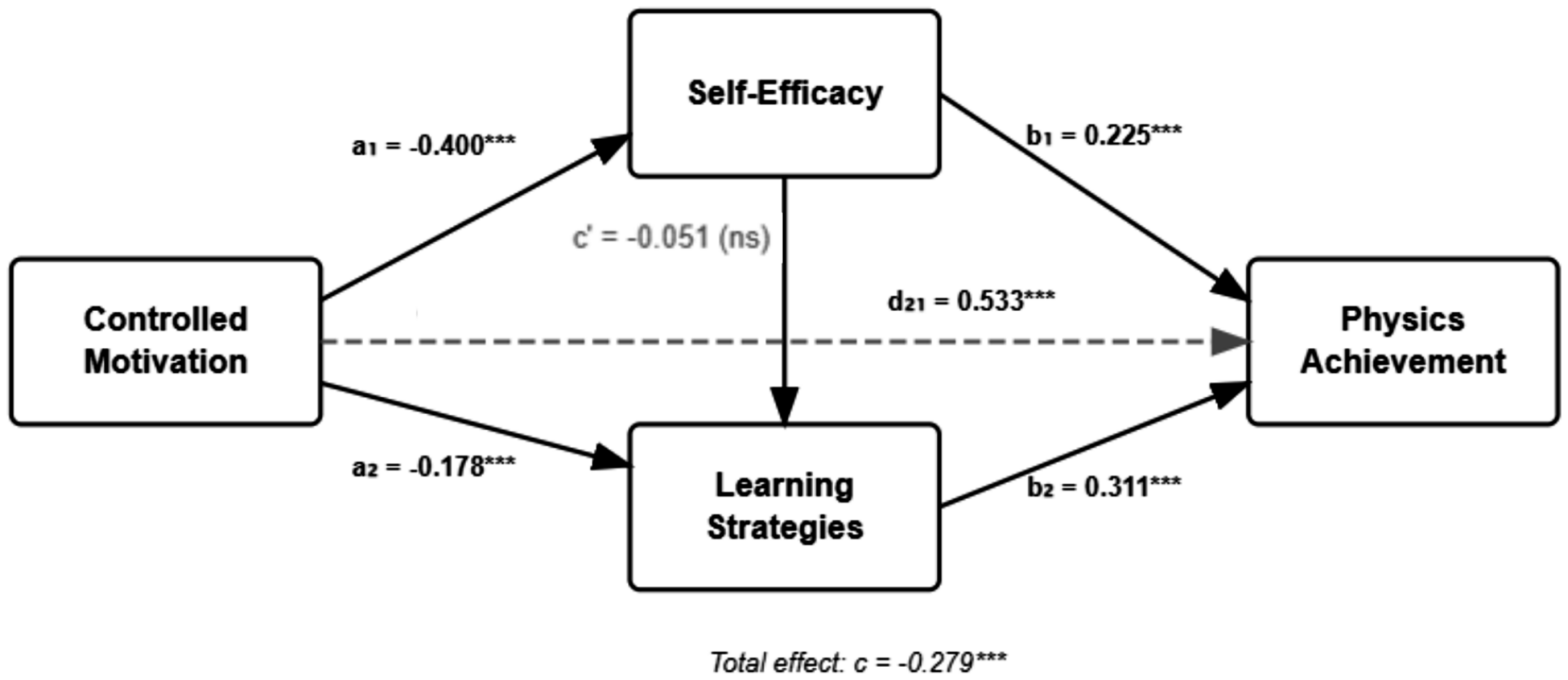
Serial mediation model with standardized path coefficients. Standardized regression coefficients are shown for each path. The dashed line represents the non-significant direct effect after controlling for mediators. *p* < 0.001.

The hypothesized serial mediation model was tested using PROCESS Model 6 with 10,000 bootstrap samples. Figure 1 presents the complete model with standardized path coefficients, and Table 4 presents the detailed results of all pathways.

**Table 4:** Path coefficients for serial mediation model.

Path	*B*	*SE*	*t*	*p*	*95% CI*	*β*
Direct effects						
Controlled motivation → Self-efficacy (a₁)	−1.142	0.125	−9.13	<0.001	[−1.388, −0.896]	−0.400
Controlled motivation → Learning strategies (a₂)	−0.648	0.162	−4.00	<0.001	[−0.967, −0.329]	−0.178
Self-efficacy → Learning strategies (d₂₁)	0.682	0.048	14.21	<0.001	[0.588, 0.776]	0.533
Self-efficacy → Physics achievement (b₁)	0.152	0.032	4.75	<0.001	[0.089, 0.215]	0.225
Learning strategies → Physics achievement (b₂)	0.164	0.025	6.56	<0.001	[0.115, 0.213]	0.311
Controlled motivation → Physics achievement (c’)	−0.098	0.089	−1.10	0.272	[−0.273, 0.077]	−0.051
Total effect						
Controlled motivation → Physics achievement (c)	−0.537	0.086	−6.25	<0.001	[−0.706, −0.368]	−0.279

*N* = 496. B = unstandardized coefficient; β = standardized coefficient; SE = standard error; CI = confidence interval.

#### 3.3.1 Direct pathways

The analysis revealed significant effects for all hypothesized direct pathways in the model. Controlled motivation negatively predicted self-efficacy (*a₁* = −1.142, *SE* = 0.125, *t* = −9.13, *p* < 0.001, *95% CI* [−1.388, −0.896]), indicating that each one-unit increase in controlled motivation was associated with a 1.14-point decrease in self-efficacy. Controlled motivation also negatively predicted learning strategies (*a₂* = −0.648, *SE* = 0.162, *t* = −4.00, *p* < 0.001, *95% CI* [−0.967, −0.329]), though this effect was somewhat smaller in magnitude.

Self-efficacy positively predicted learning strategies (*d₂₁* = 0.682, *SE* = 0.048, *t* = 14.21, *p* < 0.001, *95% CI* [0.588, 0.776]), demonstrating that students with stronger efficacy beliefs employed more effective learning strategies even after controlling for controlled motivation. This represents a moderate to large effect, with each one-point increase in self-efficacy associated with a 0.68-point increase in learning strategies. Self-efficacy also positively predicted physics achievement (*b₁* = 0.152, *SE* = 0.032, *t* = 4.75, *p* < 0.001, *95% CI* [0.089, 0.215]), indicating that efficacy beliefs directly contribute to performance beyond their effects on learning strategies.

Finally, learning strategies positively predicted physics achievement (*b₂* = 0.164, *SE* = 0.025, *t* = 6.56, *p* < 0.001, *95% CI* [0.115, 0.213]), confirming that strategy use has direct performance consequences. This effect remained significant even after controlling for both controlled motivation and self-efficacy, demonstrating learning strategies’ unique contribution to achievement (See Table 4).

#### 3.3.2 Total and direct effects

The total effect of controlled motivation on physics achievement was significant and negative (*c* = −0.537, *SE* = 0.086, *t* = −6.25, *p* < 0.001, *95% CI* [−0.706, −0.368]), indicating that students with higher controlled motivation achieved lower physics scores. This represents a moderate effect size (*β* = −0.279), suggesting that approximately 8% of variance in physics achievement is attributable to controlled motivation quality.

Critically, after including self-efficacy and learning strategies as mediators, the direct effect of controlled motivation on physics achievement became non-significant (*c’* = −0.098, *SE* = 0.089, *t* = −1.10, *p* = 0.272, *95% CI* [−0.273, 0.077]). This pattern indicates complete mediation, wherein controlled motivation’s influence on achievement operates entirely through its effects on self-efficacy beliefs and learning strategy use rather than through direct pathways.

#### 3.3.3 Indirect effects

Table 5 presents the specific indirect effects estimated through bootstrap procedures. Three indirect pathways were examined, each representing a distinct mechanism through which controlled motivation might influence physics achievement.

**Table 5:** Specific indirect effects in serial mediation model.

Indirect effect	Effect	Boot *SE*	Boot *95% CI*	*PM*
Ind1: CM → SE → PA (a₁b₁)	−0.174	0.042	[−0.261, −0.094]	32.4%
Ind2: CM → LS → PA (a₂b₂)	−0.106	0.031	[−0.172, −0.048]	19.7%
Ind3: CM → SE → LS → PA (a₁d₂₁b₂)	−0.128	0.025	[−0.181, −0.082]	23.8%
Total indirect effect	−0.408	0.058	[−0.526, −0.296]	76.0%

*N* = 496. CM = Controlled Motivation; SE = Self-Efficacy; LS = Learning Strategies; PA = Physics Achievement. Boot SE = bootstrap standard error; Boot 95% CI = bias-corrected bootstrap 95% confidence interval based on 10,000 bootstrap samples; PM = proportion mediated (indirect effect/total effect × 100%).

The first indirect effect through self-efficacy alone (Ind1: controlled motivation → self-efficacy → physics achievement) was significant (*effect* = −0.174, *SE* = 0.042, *95% CI* [−0.261, −0.094]), accounting for 32.4% of the total effect. This indicates that controlled motivation undermines achievement substantially through its erosion of students’ confidence in their physics capabilities. The second indirect effect through learning strategies alone (Ind2: controlled motivation → learning strategies → physics achievement) was also significant (*effect* = −0.106, *SE* = 0.031, *95% CI* [−0.172, −0.048]), accounting for 19.7% of the total effect. This suggests that controlled motivation directly impairs strategy use, independent of its effects on self-efficacy. Most importantly for the current theoretical framework, the serial mediation pathway (Ind3: controlled motivation → self-efficacy → learning strategies → physics achievement) was significant (*effect* = −0.128, *SE* = 0.025, *95% CI* [−0.181, −0.082]), accounting for 23.8% of the total effect. This finding provides strong evidence for the hypothesized sequential process: controlled motivation undermines self-efficacy beliefs, weakened efficacy beliefs lead to less effective learning strategy use, and ineffective strategies ultimately result in poorer achievement. The significance of this serial pathway confirms that the influence of motivational quality on performance operates through a cascading sequence of psychological and behavioral mechanisms rather than through independent parallel processes.

The total indirect effect across all three pathways was substantial (*effect* = −0.408, *SE* = 0.058, *95% CI* [−0.526, −0.296]), accounting for 76.0% of controlled motivation’s total influence on achievement. Combined with the non-significant direct effect, these results indicate complete mediation and demonstrate that controlled motivation’s detrimental effects on physics achievement are entirely explained by its sequential impacts on self-efficacy beliefs and learning strategy use.

## 4 Discussion

### 4.1 Overview of key findings

This study examined how controlled motivation influences physics achievement in Chinese high school students preparing for the Gaokao examination. The results provide strong evidence for a sequential psychological process. Controlled motivation, characterized by external pressure and learning anxiety, undermines physics achievement through cascading effects on self-efficacy beliefs and learning strategy use. The serial mediation pathway accounted for approximately 24% of the total effect. When combined with two parallel indirect pathways, indirect effects explained 76% of controlled motivation’s total influence on achievement, indicating that indirect effects account for the substantial majority of the total effect, with the direct effect becoming statistically non-significant.

### 4.2 The association between controlled motivation and achievement

The negative association between controlled motivation and physics achievement (*β* = −0.279, *p* < 0.001) aligns with self-determination theory research documenting correlations between extrinsic motivation pressures and academic outcomes (Deci and Ryan, 2016; Vansteenkiste et al., 2004). Recent meta-analytic evidence provides robust support for this relationship. Howard et al. (2021) synthesized 344 studies involving 223,209 participants and found that external regulation was not associated with performance but was linked to decreased well-being. Lee (2024) meta-analysis reported a small negative effect of controlled motivation on goal attainment (*r* = −0.13). More recently, Santana-Monagas et al. (2025) used rigorous RI-CLPM methodology to demonstrate negative cross-lagged effects of controlled motivation on mathematics achievement. The current study extends this literature by testing specific psychological mechanisms that may account for this relationship. The non-significant direct effect (*c’* = −0.051, *p* = 0.272) after statistically controlling for mediators suggests that controlled motivation’s association with achievement may operate primarily through motivational and behavioral pathways rather than through unmeasured direct mechanisms. However, the cross-sectional design prevents definitive causal inference about directional relationships.

This pattern has important theoretical implications while requiring cautious interpretation. In high-stakes examination contexts such as China’s Gaokao system, students experiencing high controlled motivation demonstrate lower performance. The mediation analysis suggests this relationship may reflect a process wherein pressure and anxiety erode psychological and cognitive resources necessary for effective learning. However, alternative explanations remain plausible. For instance, students who perform poorly may subsequently develop greater anxiety and external pressure, representing a reverse causal pathway not testable with cross-sectional data. Longitudinal research measuring these constructs across multiple waves would clarify temporal sequencing and provide stronger evidence for the proposed causal mechanisms.

### 4.3 Self-efficacy as a critical mediator

Self-efficacy emerged as the most important individual mediator, with its indirect pathway accounting for 32.4% of the total effect. This demonstrates that controlled motivation’s primary impact operates through erosion of students’ confidence in their physics capabilities. The strong negative relationship between controlled motivation and self-efficacy (*β* = −0.400, *p* < 0.001) reflects multiple psychological processes. Students experiencing high external pressure develop negative mastery experiences, as they attribute successes to external factors rather than personal capability (Bandura et al., 1999). Performance anxiety interferes with task execution, creating failure experiences that further undermine efficacy beliefs (Grant et al., 2011). These processes establish self-perpetuating cycles wherein controlled motivation generates anxiety, anxiety impairs performance, and poor performance confirms doubts about capability. The mediating role of self-efficacy is well-documented across educational contexts. Honicke and Broadbent's (2016) systematic review of 59 studies confirmed that academic self-efficacy strongly predicts academic performance. Recent cross-lagged panel analyses further demonstrate positive reciprocal relationships between self-efficacy, autonomous motivation, and academic achievement in secondary school students, with self-efficacy serving as both predictor and mediator of academic outcomes (Li et al., 2024).

The relationship between self-efficacy and physics achievement (*β* = 0.225, *p* < 0.001) remained significant after controlling for learning strategies. This suggests that self-efficacy influences achievement through multiple pathways beyond strategy use. Efficacy beliefs affect persistence in the face of difficulty, cognitive engagement during learning, and interpretation of challenging problems as opportunities rather than threats (Honicke and Broadbent, 2016). In physics, where complex conceptual understanding and flexible problem-solving are required, students with strong efficacy beliefs invest greater cognitive effort in understanding underlying principles rather than memorizing procedures (Nurulwati et al., 2020).

Gender differences in self-efficacy merit discussion. Male students reported higher confidence than female students (*t* = 3.42, *p* < 0.001, *d* = 0.32), reflecting documented disparities linked to gender stereotypes about STEM aptitude (Areepattamannil et al., 2011). Gender differences did not emerge for controlled motivation, learning strategies, or achievement itself. This suggests that gender disparities in physics operate primarily through self-efficacy beliefs rather than differential motivation or strategy use. This finding highlights the importance of interventions explicitly addressing gender-based efficacy gaps.

### 4.4 Learning strategies as behavioral manifestations of efficacy

The strong relationship between self-efficacy and learning strategies (*β* = 0.533, *p* < 0.001) demonstrates that efficacy beliefs translate into concrete behavioral changes. Students with strong confidence in their physics capabilities employ sophisticated cognitive strategies including elaboration, organization, and information integration. Students with weak efficacy beliefs adopt surface strategies emphasizing rote memorization (Gürcan, 2005). This relationship remained robust after controlling for controlled motivation, indicating that self-efficacy distinctly shapes strategy use independent of motivational pressures.

The indirect effect through learning strategies alone (*a₂b₂* = −0.106, 19.7% of total effect) reveals that controlled motivation directly constrains strategy use apart from its effects on self-efficacy. Students experiencing high anxiety and pressure adopt defensive strategies focused on immediate performance rather than deep understanding (Roderick and Engel, 2001). The intense preparation environment may also promote maladaptive behavioral patterns. Students facing examination pressure often adopt prolonged sedentary study sessions, though evidence regarding whether brief physical exercise breaks enhance cognitive performance during such sessions remains mixed (Li et al., 2022). When examination pressure is imminent and anxiety is high, students gravitate toward formula memorization and procedural routines. These strategies provide short-term performance gains but sacrifice the conceptual understanding necessary for flexible problem-solving.

The positive effect of learning strategies on physics achievement (*β* = 0.311, *p* < 0.001) was among the largest in the model. This underscores that strategy quality represents a critical determinant of performance. Deep learning strategies that encourage connection-making, conceptual organization, and elaboration prove essential for mastering abstract physics concepts and applying principles flexibly to novel problems (Cavallo et al., 2003). Surface strategies enable students to reproduce memorized procedures on routine problems but provide inadequate preparation for the conceptual reasoning demanded by Gaokao physics examinations. This pattern aligns with extensive research on self-regulated learning. Diseth (2011) found strong relations between motivational variables (self-efficacy/goal orientations) and deep/surface learning strategies, with path analyses showing structural relations wherein self-efficacy predicts both learning strategies and subsequent achievement. In medical education contexts, metacognitive learning strategies have been shown to mediate the effect of self-efficacy on academic performance (Hayat et al., 2020). Effective strategy implementation requires sustained self-regulatory capacity, and recent evidence indicates that behavioral regulation capacities may be influenced by broader factors including lifestyle patterns (Li et al., 2025).

### 4.5 Serial mediation: evidence for cascading effects

The significant serial mediation pathway (*a₁d₂₁b₂* = −0.128, 23.8% of total effect) represents a key theoretical contribution. This pathway demonstrates that controlled motivation’s influence operates as a causal sequence rather than as independent parallel processes. The mechanism unfolds sequentially. External pressure and anxiety undermine self-efficacy. Weakened efficacy beliefs lead students to adopt less effective learning strategies. Ineffective strategies ultimately result in performance decline.

This sequential arrangement has important implications for intervention design. The serial pathway’s significance suggests that breaking any link in the sequence could attenuate controlled motivation’s negative effects. Interventions targeting efficacy beliefs may prove particularly effective because efficacy operates at the beginning of the causal chain and influences both subsequent strategy use and direct achievement effects. By enhancing students’ confidence despite external pressure, educators could simultaneously improve strategy use and strengthen persistence through difficulties.

The pattern observed here approximates what Baron and Kenny (1986) termed complete mediation, wherein the direct effect becomes non-significant after including mediators. However, two qualifications warrant emphasis. First, the direct effect estimate (*c’* = −0.098) remained negative though not statistically significant, suggesting that unexplained variance may remain. The *95% CI* [−0.273, 0.077] includes both negative and near-zero values, indicating uncertainty about whether the true direct effect is exactly zero or merely small. Second, statistical non-significance does not prove the absence of an effect, only that available data do not provide sufficient evidence to reject the null hypothesis. Thus, we characterize the mediation pattern as substantial rather than claiming absolute complete mediation.

### 4.6 Contextual implications for high-stakes testing

These findings must be understood within China’s educational context. The Gaokao examination represents an extraordinarily high-stakes assessment that determines university admission and career trajectories (Wang, 1997). Recent comprehensive analyses document the examination’s pervasive influence. Jia and Li (2024) describe the Gaokao as a “centralized tournament” in which more than 10 million students compete annually, with only 5% gaining admission to top 100 universities. Their analysis reveals that four years of elite higher education yields a 40% income premium, with another 40% premium for top-tier university graduates. Yu et al. (2025) found that 76% of Chinese students experience negative mood due to academic stress, with over 71% unable to identify their true interests by graduation, suggesting that controlled motivation dominates student experience in this high-stakes context. This creates intense external pressure from parents, teachers, peers, and societal expectations.

The academic tracking system produced expected patterns. Advanced class students reported lower controlled motivation, higher self-efficacy, more effective learning strategies, and superior achievement compared to key and regular class students. These differences likely reflect both selection effects and treatment effects. Higher-achieving students tracked into advanced classes may experience less pressure despite high standards. Advanced classes may also provide more supportive, less pressure-focused instructional environments. The tracking system appears to create multiple pressures for regular and key class students who may experience both placement-based stigma and inadequate instructional resources.

### 4.7 Theoretical advances

This study extends prior research in several ways. Previous research documented that autonomous motivation predicts better achievement than controlled motivation (Deci and Ryan, 2000; Mouratidis et al., 2021) but less thoroughly investigated specific psychological mechanisms through which controlled motivation impairs performance. By simultaneously measuring self-efficacy and learning strategies as sequential mediators, this study clarifies that controlled motivation’s effects operate through motivation-belief-behavior chains rather than single direct pathways.

The serial mediation framework integrates self-determination theory, social cognitive theory, and self-regulated learning theory into a unified explanatory model. Self-determination theory explains why controlled motivation is problematic. Social cognitive theory explains how controlled motivation erodes efficacy beliefs. Self-regulated learning theory explains how efficacy beliefs shape strategy use. This theoretical integration proves more parsimonious and empirically supported than examining these constructs in isolation.

The finding that controlled motivation pathways account for 76% of variance in the motivation-achievement relationship quantifies the importance of sequential psychological processes. Understanding achievement requires attending not just to final grades but to the psychological and behavioral sequences through which motivation translates into performance.

### 4.8 Limitations and future directions

Several limitations warrant acknowledgment. First, this study employed cross-sectional data, precluding causal inference despite the theoretical basis for the hypothesized model. Longitudinal designs measuring motivation, efficacy, and strategies across multiple time points would strengthen causal claims and enable examination of whether changes in controlled motivation predict subsequent changes in efficacy and strategies. Second, the study focused on physics in Chinese high schools, limiting generalizability to other subjects, educational levels, or countries. Physics represents a demanding subject with particular relevance to STEM career pathways, but motivational mechanisms might differ for less conceptually demanding subjects or in educational systems with lower examination pressure. Third, we measured learning strategies through student self-report. Direct observation of study behaviors or analysis of study materials could provide more objective assessment of strategy implementation. Self-reported strategy use may reflect perceived sophistication more than actual practice. Fourth, the study did not examine potential moderators of mediation pathways such as family socioeconomic status, parental education, or classroom climate. These contextual factors may strengthen or weaken the proposed relationships. Future research should investigate whether classroom autonomy-supportive practices buffer against controlled motivation’s detrimental effects by maintaining efficacy and strategy use despite external pressure (Deci and Ryan, 2008). Fifth, this study examined motivation operationalized specifically as external pressure and learning anxiety rather than assessing the complete motivation spectrum. Future research incorporating multidimensional motivation measures would clarify how different motivation types combine to influence achievement. Sixth, this study employed a convenience sampling approach, recruiting participants from three schools in one city. While our stratified sampling ensured representation across academic tracks within these schools, the non-random selection limits generalizability. Replication studies using probability sampling from diverse geographic regions across China are needed to establish whether these findings extend to the broader population of Chinese high school physics students. Additionally, future research should examine whether these relationships hold in other educational systems with different examination structures and cultural contexts. Seventh, we did not assess lifestyle and behavioral factors that may moderate these relationships. Recent evidence indicates that 24-h movement behaviors—including physical activity, screen time, and sleep duration—significantly influence attention, self-regulation, and academic engagement (Li et al., 2024). Future research should investigate whether such factors moderate the relationships between controlled motivation, self-efficacy, and learning strategies, as students experiencing high examination pressure may adopt unhealthy behavioral patterns that further compromise cognitive and motivational resources.

### 4.9 Practical implications

These findings suggest several intervention points. At the classroom level, teachers can work to maintain or enhance student self-efficacy despite external pressure. This can be achieved by providing achievable challenges with explicit feedback linking performance to capability rather than effort or luck (Bandura et al., 1999). Efficacy-building interventions might emphasize mastery experiences through appropriately calibrated tasks, persuasive feedback highlighting capability growth, and modeling by capable peers. Second, explicit strategy instruction targeting deep learning approaches could help students translate maintained efficacy into effective study behaviors. Many students facing examination pressure default to surface strategies without conscious awareness of alternatives. Systematic instruction in elaboration, organization, concept mapping, and active problem-solving could interrupt the pathway from anxiety to ineffective strategies (Harlen and Deakin Crick, 2003). Third, at the systemic level, reducing unnecessary external pressure while maintaining high academic standards could preserve motivation quality. The complete mediation pattern suggests that pressure reduction could substantially improve achievement by preserving efficacy beliefs and strategy quality. Alternative assessment approaches emphasizing growth, mastery, and learning rather than high-stakes competition might reduce controlled motivation while maintaining academic rigor (Wise and Kingsbury, 2016).

## 5 Conclusion

This study examined how controlled motivation influences physics achievement among Chinese high school students through sequential psychological and behavioral mechanisms. Using serial mediation analysis, we demonstrated that controlled motivation, characterized by external pressure and learning anxiety, undermines achievement by eroding self-efficacy beliefs, which subsequently reduces adoption of effective learning strategies. The serial mediation pathway accounted for 24% of the total effect, with complete mediation indicating that controlled motivation’s effects operate entirely through identified psychological and behavioral mechanisms.

These findings advance theoretical understanding of motivation’s effects in high-stakes educational contexts by identifying specific intervention points. Maintaining self-efficacy despite pressure could preserve strategic learning approaches and academic performance. The research highlights that in examination-intensive educational systems, addressing motivational pressures requires not just reducing external demands, which may prove impossible, but rather building psychological resilience through efficacy enhancement and strategic learning support. Future research employing longitudinal designs across diverse educational contexts will further clarify these relationships and expand understanding of how motivational processes shape achievement trajectories.

## Data Availability

The raw data supporting the conclusions of this article will be made available by the authors, without undue reservation.
